# Chest-abdomen-pelvis CT for staging in cancer patients: dose effectiveness and image quality using automated attenuation-based tube potential selection

**DOI:** 10.1186/s40644-014-0028-7

**Published:** 2014-09-02

**Authors:** Martin Beeres, Marcus Römer, Boris Bodelle, Clara Lee, Tatjana Gruber-Rouh, Emmanuel Mbalisike, Josef M Kerl, Julian L Wichmann, Boris Schulz, Thomas J Vogl, Ralf W Bauer

**Affiliations:** 1 Department of Diagnostic and Interventional Radiology, Clinic of the Goethe University, Theodor-Stern-Kai 7, Frankfurt, 60590, Germany

**Keywords:** Multidetector computed tomography, Cancer, Cancer staging, Neoplasms

## Abstract

**Background:**

Evaluation of automated attenuation-based tube potential selection and its impact on image quality and radiation dose in CT (computed tomography) examinations for cancer staging.

**Methods:**

A total of 110 (59 men, 51 women) patients underwent chest-abdomen-pelvis CT examinations; 55 using a fixed tube potential of 120 kV/current of 210 Reference mAs (using CareDose4D), and 55 using automated attenuation-based tube potential selection (CAREkV) also using a current of 210 Reference mAs.

This evaluation was performed as a single-centre, observer-blinded retrospective analysis. Image quality was assessed by two readers in consensus. Attenuation, image noise, signal-to-noise ratio (SNR), and contrast-to-noise ratio (CNR) were measured or calculated for objective image evaluation. For the evaluation of radiation exposure, dose-length-product (DLP) values were compared and Size-specific dose estimates (SSDE) values were calculated.

**Results:**

Diagnostic image quality was obtained from all patients. The median DLP (703.5 mGy · cm, range 390–2203 mGy · cm) was 7.9% lower when using the algorithm compared with the standard 120 kV protocol (median 756 mGy · cm, range 345–2267 mGy · cm). A reduction in potential to 100 kV occurred in 32 cases; therefore, these patients received significantly lower radiation exposure compared with the 120 kV protocol.

**Conclusion:**

Automated attenuation-based tube potential selection produces good diagnostic image quality in chest-abdomen-pelvis CT and reduces the patient’s overall radiation dose by 7.9% compared to the standard 120 kV protocol.

## Background

In the clinic, the number of computed tomography (CT) examinations is increasing steadily [1],[2]. Compared with most other imaging modalities, CT imaging involves the use of increased radiation exposure [3]. In CT examinations as well as in all other examination modalities, the ‘as low as reasonably achievable’ (ALARA) rule has to be considered, especially when radiation is applied to the patient. However, not only are the number of CT examinations increasing, the examination volume (e.g. chest-abdomen-pelvis examination) is increasing, too [3]. In terms of staging cancer, the patient’s cumulative radiation exposure might cause problems in the future [3]. In some cases, cancer is already the limiting disease, and therefore the benefits of staging cancer using radiation exposure outweigh the risks [4]. However, some cancer patients—especially those in the early stages—will live long enough that the long-term effects of radiation become significant [5]. Many techniques are already in use to minimise the radiation exposure of CT examinations: Automated attenuation-based tube current and voltage modulation as well as noise reduction filters and iterative reconstruction algorithms are current options [6],[7].

Since dose modulation software for the adjustment of tube current was introduced, it has been used routinely all over the world and remains an important invention for reducing the radiation dose from imaging techniques [6],[8]–[11]. Automated X-ray tube potential selection is also providing clinical radiologists and technicians another opportunity to adapt the radiation dose of the CT examination to the requirements of the specific body region [12]–[16].

The rationale behind automated tube potential selection is that the contrast can be improved using lower X-ray tube potential, because low-energy X-rays are better absorbed than high-energy X-rays [17]. For larger patients, the tube potential sometimes has to be adjusted to higher levels due to increased absorption occurring at a low tube potential [17].

Since automated attenuation-based tube potential selection was introduced in 2011, the technique has helped to lower radiation exposure, presumably as well as the automated tube current modulation has done, and continues to do so [14]. A limited number of studies have investigated radiation exposure while using automated tube potential selection [12]–[16],[18]. One of the first trials reported using automated tube potential selection was performed for the imaging of the great vessels; in this case, it was possible to lower radiation exposure by 25.1% [18]. This may be too much of a reduction for chest-abdomen-pelvic CT examinations, so the aim of our study was to evaluate automated tube potential selection for chest-abdomen-pelvic CT examinations carried out for staging reasons in cancer patients.

## Methods

### Patients

The study was performed as a single-centre, observer-blind study. The Institutional Review Board of our University Clinic (Goethe University Clinic, Frankfurt, Germany) approved this study; written informed consent requirement was waived since CAREkV is routinely used in all patients undergoing clinically indicated CT in our department. The data from consecutive unselected patients who underwent clinically indicated chest-abdomen-pelvis staging CT between January 2011 and March 2012 were analysed. The general exclusion criteria for contrast-enhanced CT included impaired renal function (estimated glomerular filtration rate <60 mL/min, calculated by creatinine blood level and patient age), hyperthyroidism, as well as hypersensitivity to iodine contrast media.

A total of 110 patients (59 men and 51 women, median age 65 [range 35–95 years]) underwent a 128-slice chest-abdomen-pelvis CT examination: 55 using a fixed tube potential of 120 kV/210 Ref.mAs, and 55 using automated attenuation-based tube potential selection that selected the tube current based on the attenuation profile of the topogram (CAREkV), adjusted to a predefined image quality of 120 kV/210 Ref.mAs (Table 1). Chest-abdomen-pelvis CT examinations were performed for staging reasons in all patients. Patient populations were paired regarding sex, age, body size and habitus. The cancer-staging examinations were performed on a broad range of cancer origins; the evaluated diseases are listed in detail in Table 2.

**Table 1:** Study population and examination parameters

	**Group 1**	**Group 2**
Imaging mode	Single-source	Single-source
Slice · collimation (mm)	128 · 0.6	128 · 0.6
Pitch	1.2	1.2
kV/ref.mAs	120/210 (CarekV)	120/210 (CareDose4D)
Patients 100 kV	32	
Patients 120 kV	17	55
Patients 140 kV	6	

**Table 2:** Study population – indications and pathologies

**Patients**	**Diagnosis**
16	Colorectal cancer
15	Lymphoma
14	Malignant melanoma
14	Breast cancer
9	Non-small-cell lung carcinoma (NSCLC)
6	Oral cancer
6	Hepatocellular carcinoma
5	Renal cell carcinoma
4	Urothelial cell carcinoma
2	Pancreas Carcinoma
2	Cholangiocarcinoma
2	Small cell lung cancer (SCLC)
2	Seminoma
2	Gastric cancer
2	Hypopharyngeal cancer
2	Tonsil cancer
1	Leiomyosarcoma
1	Synovial sarcoma
1	Choriocarcinoma
1	Ovarian cancer
1	Ewing sarcoma
1	Osteosarcoma
1	Merkel cell carcinoma

### Automated attenuation-based kV selection

**Figure 1:**
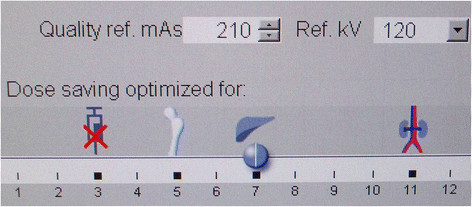
Care-kV settings for the experimental group (group 1).

The automated tube potential selection CAREkV (Siemens Healthcare, Forchheim, Germany) selects the optimum tube potential for the diagnostic region to be examined. Different settings can be adjusted during the imaging protocol set up. Adjustment can be made by using a 12-point scale tool as seen in Figure 1. This is useful, because for a high-contrast situation such as vascular imaging, lowering of the kV leads to higher absorption and higher attenuation values of the examined vascular structures. After this ‘pre-set’ procedure, depending on the body region and the anatomic structure to be examined, the tube potential is selected by the patient’s topogram or scout. This is similar to the tube current modulation already in use in nearly every radiology department around the world (e.g., CareDose4D, Siemens; Auto-mA, GE; DOM [DoseModulation], Philips; Real E.C., Toshiba).

The automatically selected kV remained stable throughout the whole examination. After that, the corresponding current, in mAs, was calculated. This was selected in order to attain the user-given image quality (e.g., 120 kV, 210 Ref.mAs). The user of the instrument provided the machine with a preferred image quality, which mostly depended on the standards in the particular radiology department. Then, the machine automatically selected the voltage and current, according to the type of examination being performed (e.g., vascular, bone, parenchymal, Figure 1). In our study, we used the algorithm for parenchymal organs because the CT examinations were performed for cancer staging reasons (Figure 1).

### CT protocol

CT examinations were performed on the experimental group using a 128-slice CT machine (SOMATOM Definition Flash, Siemens, Forchheim, Germany), using automated attenuation-based tube potential selection (CAREkV). In the baseline group (conventional group) without CAREkV, only CareDose4D and a fixed tube potential of 120 kV were used.

Before the examination, the patients were enterally administered contrast media (Micropaque, Guerbet, Villepinte, France) and during the examination an intravenous contrast material, containing 1 ml/kg of iodinated contrast material (Ultravist 370, Bayer-Schering, Germany), followed by a saline chaser of 40 mL.

Contrast material, as well as saline flush, was injected at 2 mL/s into an antecubital vein using a double-syringe power injector (CT2, Medtron, Saarbruecken, Germany). A fixed delay of 70 seconds post injection was used in order to obtain venous contrast. All CT examinations were performed in a cranio-caudal direction starting from the upper thorax aperture down to the femoral ligaments, at a collimation of 128 · 0.6 mm, pitch 1.2, and rotation time of 0.5 seconds.

CT examinations were performed as follows:

 Group 1 (experimental group) – 120 kV (using CAREkV – where kV can be automatically adapted to any of the following values: 80, 100, 120, and 140 kV), 210 Ref.mAs.

 Group 2 (conventional group) – 120 kV (fixed), 210 Ref.mAs (using CareDose4D);

### CT data reconstruction

For fast overviewing, images were reconstructed in 5-mm slice thickness using a 5-mm increment. For further evaluation, data were reconstructed with a slice thickness of 2 mm and increment of 1.5 mm using a medium-smooth soft-tissue convolution kernel (B30f) for parenchymal analysis. A hard convolution kernel was used for the analysis of bones and lungs (B70f). For detailed analysis and post-processing, images were transferred to an external workstation (SyngoVia, Multi-Modality Workplace, Siemens Healthcare, Forchheim, Germany).

### Radiation dose estimations

Effective mAs, kV, CTDIvol and DLP values were evaluated using the patient protocol saved in our PACS system after each examination was performed. However, CTDIvol is only a measurement of scanner output and does not include information about patient size; therefore, additional SSDE values were calculated according to the AAPM (American Association of Physicists in Medicine) Report 204 [19]. For the calculation of the SSDE, patient dimensions such as anteroposterior (AP) thickness at the midline (measured from axial CT images) and lateral (LAT) width were determined from the topogram (Tables 3 and 4). Once the patient size is determined, f-size can be found from the appropriate table in the AAPM Report 204 or computed by use of a mathematical equation, as we did in this study [19],[20]. For each patient, AP and LAT dimensions, as well as SSDE values, were calculated according to the study by Christner et al. [20].

**Table 3:** Overview of patient characteristics

	**Group 1**	**Group 2**	**p-Value:**
			**Group 1 vs. Group 2**
Patients	55	55	
Male	27	32	
Female	28	23	
Age (years)	61 (20–82)	59 (21–88)	
Body diameter: transverse (cm)	24.7 (15.1–40.9)	24.2 (15.6–44.6)	0.52
Body diameter: lateral (cm)	34.4 (28.1–47.8)	33.1 (19.6–49.6)	0.33
Scanning range (cm)	66.1 (50.8–76.4)	65.5 (57.1–75.4)	0.5
CTDIvol (mGy · cm)	10.5 (5.8–31.7)	11.4 (6.0–36.0)	0.5
SNR	26.4 (3.9 – 58.9)	32.2 (19 – 52.3)	< 0.01
CNR	17.7 (3.7 – 42.5)	19.8 (12.1 – 34.1)	0.02
SSDE	13.3 (9.7 – 28.6)	14.8 (9.4 – 28.9)	0.6

**Table 4:** Detailed overview of the different examination protocols

		**Patients**	**Noise**	**SSDE**	**Effective mAs**	**p-Value**	**p-Value**	**p-Value**
						**Overall-Noise**	**Overall-SSDE**	**Overall- Eff. mAs**
						**Group 1 vs. Group 2**	**Group 1 vs. Group 2**	**Group 1 vs. Group 2**
Group 1	Patients 100 kV	32	5.4 (4.0–8.2)	11.7 (9.7–14.5)	211 (141–353)	p < 0.001	p = 0.6	p < 0.001
	Patients 120 kV	17	5.1 (3.5–11.7)	15.9 (11.9–21.8)	196 (105–285)			
	Patients 140 kV	6	6.5 (4.7–12.4)	22.4 (20.9–28.6)	216 (204–322)			
Group 2	Patients 120 kV	55	4.4 (3.1–6.7)	14.8 (9.4–28.9)	169 (89–534)			

### Image quality

Subjective image assessment was graded by two radiologists in consensus (with 2 and 5 years of experience in whole body imaging), applying a five-point scoring system: 1 = excellent: excellent definition of tumour and/or metastases, excellent delineation of the structures; 2 = good: good definition of tumour and/or metastases, minimal image noise; 3 = adequate: adequate definition of tumour and/or metastases, slight impact of image noise, sufficient for diagnosis; 4 = poor: poor definition of tumour and/or metastases, low attenuation and difficult delineation of the structures, increased image noise, diagnostic confidence reduced; 5 = unacceptable/nondiagnostic. The most probable reasons for reduced image quality were noted. Factors reducing image quality (obesity, motion, metallic artefacts, contrast medium flow-related, and contrast timing) were recorded by the radiologists.

Objective image quality (e.g., attenuation, noise, CNR) analysis was performed by one radiologist with 5 years of experience in general radiology on a regular PACS workstation (Centricity 4.2, General Electric Healthcare, Munich, Germany). The measurements were performed on several anatomic regions of the body (aorta at the level of the pulmonary trunk, both lobes of the lung, left lobe of the liver, right lobe of the liver, pancreas, spleen, kidneys, gluteus maximus muscle, bone and pre-sternally in the air). For calculation of SNR, image noise (or background noise) was determined as the standard deviation (SD) of air measured pre-sternally in front of the patient at the level of the ascending aorta. Based on these measurements, the signal-to-noise ratio (SNR) was determined according to the following equation: SNR = attenuation/background noise (Table 3). The CNR was defined as the difference in signal intensity between the venous attenuation in the vascular system (aorta) and the gluteus maximus muscle divided by the background noise. CNR was calculated as follows: CNR = [(ROIaorta – ROImuscle)/background noise]. To minimise bias from single measurements, we calculated the average of four measurements for each ROI.

Tumour and/or metastases were evaluated and measured using tumour diameters for objective evaluation and, for subjective scoring, we evaluated whether the imaging modality might have affected the tumour staging. To analyse the different habitus of each patient, the antero-posterior and the lateral diameter of the abdomen were measured at the level of the celiac trunk, and correlated to the kV that the dose modulation software had chosen for the examination (Table 5).

**Table 5:** Correlation analysis of DLP (mGy-cm) and patient diameter

		**Patients**	**DLP (mGy · cm)**	**Body diameter (mm): lateral**	**Body diameter (mm): transverse**	**Spearman correlation coefficient (rho) – lateral diameter/DLP**	**Spearman correlation coefficient (rho) – transverse diameter/DLP**
Group 1	Patients 100 kV	32	564 (390–871)	327.3 (280.7–408)	228.2 (150.7–317.2)	0.81	0.87
	Patients 120 kV	17	833 (470–1235)	351.2 (281.1–452.5)	249 (165.7–346.8)	0.80	0.85
	Patients 140 kV	6	1302 (1157–2203)	442.5 (401.5–478)	290.1 (259–409)	0.42	0.43
Group 2	Patients 120 kV	55	756 (345–2267)	330.8 (196.3–496.1)	241.9 (155.9–446.3)	0.88	0.82

### Statistical analysis

Statistical analysis was performed using dedicated software (Bias for Windows 9.14; Epsilon, Germany). Continuous variables were reported as median and range, categorical variables as frequencies or percentages.

Radiation parameters and quantitative image parameters (e.g. noise, attenuation) were tested using the Wilcoxon Mann-Whitney U test as the data were nonparametric. The relationship between patient diameter and automated kV selection was analysed using the Spearman rank order correlation test. The Chi-square (X2) test was used for categorical variables (demographic patient data). Statistical significance was defined as a p-value above 0.05.

## Results

### Radiation dose estimation

Automated attenuation-based tube potential selection resulted in a kV reduction of 120 to 100 kV in the experimental group, compared with the conventional group in 32/55 (58.2%) patients; it was kept stable at 120 kV in 17/55 (30.9%) patients and increased to 140 kV in 6/55 (10.9%) patients.

**Figure 2:**
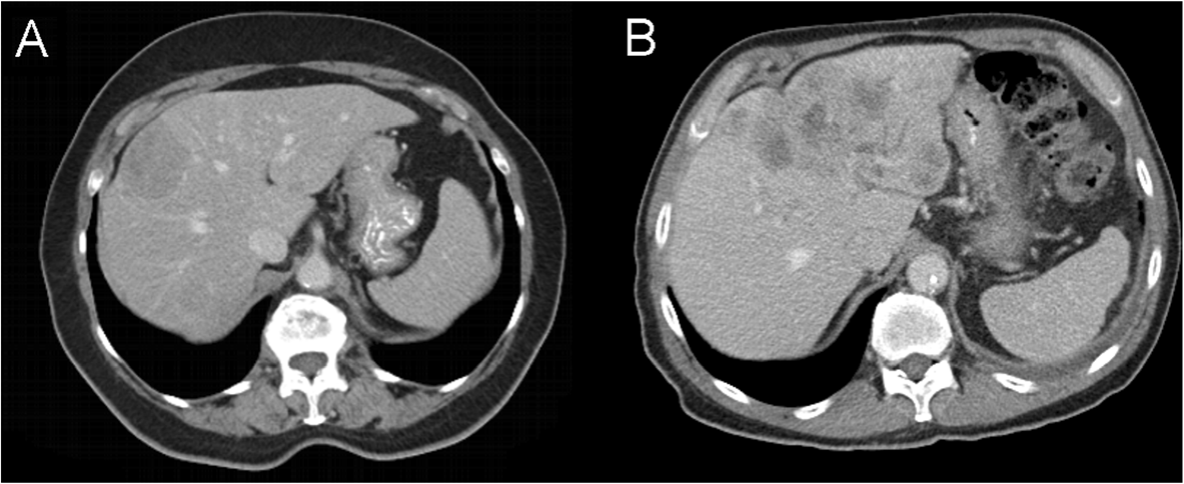
**Image quality comparison in parenchymal lesions.** Pat. **A**: Patient suffering from malignant melanoma, liver metastasis - DLP 781 mGy · cm, without CarekV, Pat. **B**: Patient suffering from colorectal cancer, liver metastasis - DLP 485 mGy · cm, using CarekV.

The median DLP in the experimental group was 564 mGy · cm (390–871 mGy · cm) at 100 kV, 833 mGy · cm (470–1235 mGy · cm) at 120 kV, and 1302 mGy · cm (1157–2203 mGy · cm) at 140 kV. The analysis between 100 and 120 kV, as well as 140 kV, showed statistical significance (Figure 2). The different values in median and range are listed in Table 3.

The difference of DLP between the groups (CAREkV vs. non-CAREkV) did not reach statistical significance (p = 0.43) (Figure 2).

Comparing our radiation exposure data from the experimental and conventional groups using SSDE calculations, it was clear that there was no significant difference (Table 3). However, the inter-group analysis showed that there was a significant difference between the 100 kV group compared to the 120 kV group (p < 0.001) and to the 140 kV group (p < 0.001) (Table 4). Between the 120 kV group and the 140 kV group there was no significant difference in SSDE values (p = 0.1).

However, this inter-group analysis should be examined with caution because there were only 6 patients in the 140 kV group. However, the results from the 100 kV group show that 32 patients were exposed to significantly less radiation, although the overall difference between the experimental and the conventional group did not reach statistical significance (SSDE p-value = 0.6; Table 3).

Patient diameter was similar in the CAREkV group and the conventional group, with a median lateral diameter of 34.4 cm (range 28.1–47.8 cm) and 33.1 cm (range 19.6–49.6 cm), respectively (Table 1).

### Image quality

Diagnostic image quality was obtained from all patients (excellent: n = 42; good: n = 9; moderate: n = 4). The reasons for moderate image quality (120 kV, n = 1; 100 kV, n = 3) were, in all cases, due to difficulties in ruling out parenchymal lesions, some because of image noise, and some because of insufficiencies concerning the contrast.

Concerning image quality in the control group, it was also rated as sufficient in all cases (excellent: n = 45; good: n = 7; moderate: n = 3).

**Figure 3:**
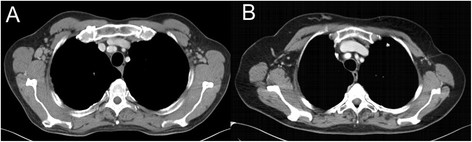
**Image quality comparison concering lymph-nodes**. Pat. **A**: Patient suffering from follicular lymphoma - DLP 644 mGy · cm, without CarekV, Pat. **B**: Patient suffering from breast cancer - DLP 599 mGy · cm, using CarekV.

**Figure 4:**
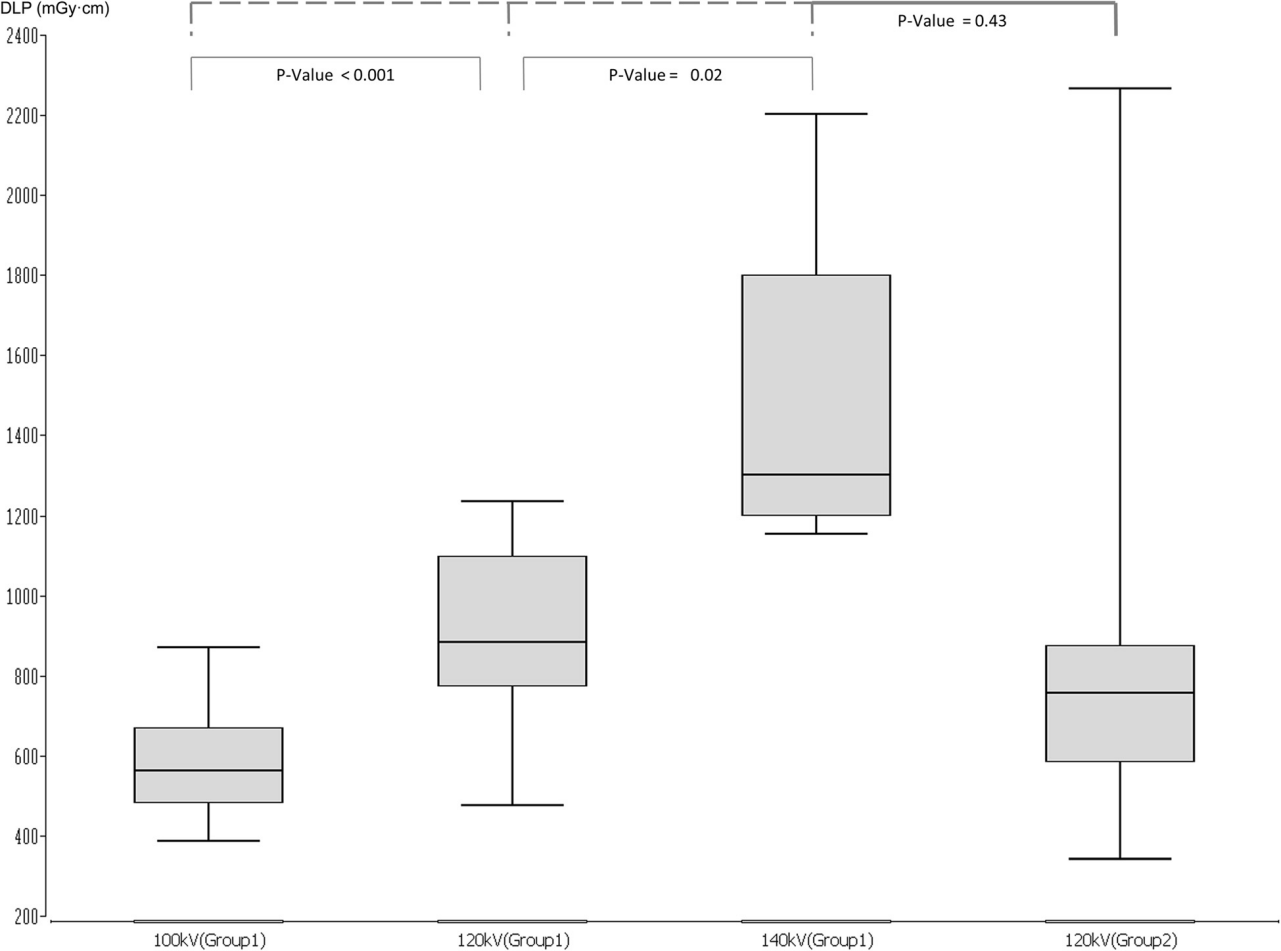
**DLP Values between the different groups.** The above p-Value is between Group 1 and 2 in general. The lower p-Values is the analysis of group 1 in the inter-group comparison.

In summary, the image quality meant that all malignancies could be ruled out sufficiently and classified correctly with respect to RECIST (Response Evaluation Criteria In Solid Tumours), without statistically significant differences between the two groups (p = 0.5) (Figures 3 and 4) [21].

## Discussion

Since the introduction of automated attenuation-based tube potential selection, most of the literature about it has been in the context of vascular imaging [18],[22]. In fact, only two studies have been published with a focus on cancer patients [15],[16].

Of course, this is not the only technique available for lowering radiation exposure, and many new techniques are in use today; automated tube-currents as well as automated tube- potential selection are only two of many components that can be modified for reducing radiation exposure. Other interesting techniques like iterative reconstruction are also in use and have been shown to have additional potential in reducing radiation exposure [6]. We wanted to evaluate whether this new technique would be valuable for the imaging of cancer patients concerning a non-stop (one-stop-shop) chest-abdominal-pelvis CT examination in a daily clinical routine. We evaluated a number of patients undergoing chest-abdomen-pelvis CT for cancer staging.

In CT, a reduction in radiation dose by lowering the tube potential results in a higher attenuation of the iodinated contrast media. However, this does not always have a positive effect, as the image noise will increase and soft-tissue contrast might be lost. To avoid this, the tube current has to be adapted to the lower kV setting (often to higher values). Therefore, the image noise will be comparable, but the radiation exposure will be lower compared to a 120 or 140 kV setting (Tables 3 and 4, Figure 2).

Winklehner et al. [18] evaluated automated attenuation-based kV selection in 40 patients for CT angiography of the aorta. In this study, an overall radiation dose reduction of 25.1% was observed, while keeping the image quality stable, when using a 120 kV protocol.

Eller et al. [15] evaluated automated attenuation-based kV selection in 100 patients. They carried out an abdominal CT examination for 52 of the patients, and a thoraco-abdominal examination for 48 patients. All examinations using automated attenuation-based kV selection resulted in a radiation dose reduction of at least 11.4%; in detail, 13.2% in the abdominal CT group and 9.5% in the thoraco-abdominal group.

Gnannt et al. [16] assessed automatic attenuation-based kV selection in 40 patients suffering from testicular cancer. In this study, a CT scan of the chest was performed in a mixed arterio-venous phase and the abdominal CT examination was carried out in the portal-venous phase of enhancement. The overall dose reduction was 12% on average. In all the studies mentioned above, there was no statistically significant worsening of subjective image quality.

In the study by Eller et al. [15], there was a dose reduction of 9.5% in thoraco-abdominal CT examination. Our data showed an overall dose reduction in 7.9% of the study population; this might be caused by different patient characteristics (e.g., body size and habitus). The tube potential in our study switched more often to 140 kV compared with the cited studies, and a tube potential of 80 kV was not automatically chosen in any case (Tables 1 and 4).

In the imaging of vessels, it is possible to examine the region of interest using a lower kV setting because of the high-contrast situation attained by the arterial phase of the contrast material. In clinical settings where parenchymal contrast is the object in question, for example, when parenchymal liver lesions have to be ruled out, automated attenuation-based tube potential selection might not lower the kV in the same aggressive manner as in the imaging of vessels (Figure 1).

Applying the algorithm to our study population, the overall radiation dose reduction was 7.9%. Comparing the DLP and SSDE values in the experimental group, it can be seen that the 100 kV patients received a significantly lower dose compared with both other groups (31 patients received an examination at the 100 kV setting, 17 at 120 kV, and only 7 at 140 kV).

In summary, these data indicate that in 31 patients, radiation exposure was significantly reduced, whereas in 24 (17 + 7) patients, radiation exposure remained the same or was not significantly increased. Keeping this in mind, more than half of the examined patients could potentially be examined with a lower radiation exposure in daily clinical routine. In a busy radiology department, sometimes it is difficult to adapt a CT examination protocol in detail to each patient, therefore, software such as CAREkV is able to improve the workflow and reduce radiation exposure.

### Limitations

Our study has some limitations. First, the overall number of patients was limited and further studies using a larger population are required. Second, we did not record the body mass index in detail, but added the patient diameter to this study as an alternative. Third, CAREkV is able to reduce the tube potential to as low as 80 kV. This tube potential, however, was not selected by CAREkV in our patient cohort. One cause for this might be the adult patient cohort, with a relatively large patient diameter on average. Also, good parenchymal contrast is needed in CT imaging for cancer staging.

## Conclusion

In summary, we showed that attenuation-based tube potential selection is a good tool for dose reduction in daily clinical routine when ‘one-stop-shop’ cancer staging is performed. It leads to a good and stable overall image quality with only a mild increase in image noise. Radiation exposure was decreased by 7.9% compared to our former standard protocol using 120 kV.

### Key points:

1. Automated attenuation-based tube potential selection is easy to use and contributes to dose reduction efforts in daily clinical routine.

2. When comparing 120 kV fixed tube potential using automated tube current modulation and automated attenuation-based tube potential selection, both lead to the same image quality.

## Competing interests

Dr. Ralf W. Bauer: Research consultant and speakers bureau, Siemens AG. Dr. J. Matthias Kerl: Research consultant and speakers bureau, Siemens AG. Prof. Dr. E. Herrmann: Consultant – F. Hoffmann-La Roche Ltd; Consultant – Novartis AG. The other authors declare that they have no competing interests.

## Authors’ contributions

Study concepts: MB, RWB, MK, BS, BB. Study design: MB, MR, BB. Data acquisition: MB, MR, BB. Quality control of data and algorithms: MB, RWB, BB, BS. Data analysis and interpretation: MB, VJ, MR, ML. Statistical analysis: MB, MR, EH. Manuscript preparation: MB, MR, RWB, TJV. Manuscript editing: MB, RWB, VJ, JW, ML. Manuscript review: TJV, VJ, RWB, MK, EH. All authors read and approved the final manuscript.
